# Eye and head movements are complementary in visual selection

**DOI:** 10.1098/rsos.160569

**Published:** 2017-01-18

**Authors:** Grayden J. F. Solman, Tom Foulsham, Alan Kingstone

**Affiliations:** 1 University of Hawai'i at Mānoa, 2530 Dole Street, Sakamaki D401, Honolulu, HI 96822-2294, USA; 2 University of Essex, Colchester, Essex, UK; 3 University of British Columbia, Vancouver, British Columbia, Canada

**Keywords:** scene viewing, embodied cognition, visual selection, head and eye movement

## Abstract

In the natural environment, visual selection is accomplished by a system of nested effectors, moving the head and body within space and the eyes within the visual field. However, it is not yet known if the principles of selection for these different effectors are the same or different. We used a novel gaze-contingent display in which an asymmetric window of visibility (a horizontal or vertical slot) was yoked to either head or eye position. Participants showed highly systematic changes in behaviour, revealing clear differences in the principles underlying selection by eye and head. Eye movements were more likely to move in the direction of visible information—horizontally when viewing with a horizontal slot, and vertically with a vertical slot. Head movements showed the opposite and complementary pattern, moving to reveal new information (e.g. vertically with a horizontal slot and vice versa). These results are consistent with a nested system in which the head favours exploration of unknown regions, while the eye exploits what can be seen with finer-scale saccades.

## 1. Introduction

During routine behaviour in naturalistic environments, we are faced with ongoing decisions about where to attend and how to orient our sensory effectors to obtain information about the world. In vision, our moment-to-moment grasp of the visual world is severely limited, with the visual field encompassing less than 180° (approximately 120° binocular), and within that range acuity falls precipitously away from the focal (foveated) area, with peripheral information severely degraded, low frequency, and having minimal chromatic sensitivity [1].

Given the importance of orienting behaviours, a large body of research has been devoted to understanding the processes that govern the selection of where and when to deploy our limited sensory resources. In vision, work on this issue has highlighted the distinction between (i) ‘covert’ selection—proceeding independently from the orienting of the receptors, and presumed to reflect shifts in neural activation to emphasize the depth and quality of processing in selected regions of the visual field, and (ii) active or ‘overt’ selection, characterized by bodily movements in service of aligning the receptors (i.e. the fovea) in the world. In naturalistic settings, active selection involves the coordinated movements of the eyes, head and trunk [2,3]. The vast bulk of research, however, has been conducted in laboratory settings where there has been a particular focus on movements of the eyes—under explicit constraints of restricted head and body movements.

As a consequence, little work has sought to evaluate potential differences in the roles and characteristics of different overt orienting systems (e.g. comparing eye-, head- or hand-based selection). Although different effectors are in many cases closely coordinated (e.g. [4–6]), several notable exceptions highlight that such coordination is by no means guaranteed. For instance, during search, locations are revisited less often when searchers must move their whole bodies through an array (when compared with search with eye movements) [7], and direct tests of memory use for eye-contingent when compared with head-contingent search have revealed a greater reliance on memory when searching with the head [8,9]. Additionally, in manually assisted search where overlapping items must be interacted with and moved, it has been shown that the motoric selection process is largely decoupled from perceptual selection [10–12].

These results give preliminary indications that *how* information is selected is an important factor in determining *what* information is selected. However, visual search tasks commonly restrict or control the stimulus features that need to be selected, whereas in naturalistic settings the features driving selection are often less clearly defined (incorporating both bottom-up featural cues as well as more complex top-down factors). A different way to investigate selection is through ‘free-viewing’ paradigms, wherein images of natural scenes are used as a proxy for the real world. Generally, participants are presented with a scene, and given a set amount of time for visual exploration—typically under instructions to either memorize or else make some broad judgement about the scene's content. Researchers can then examine the sequence of fixations (regions selected for high acuity inspection), and measure properties of these regions in order to infer the underlying causes for selection. Using these approaches, it has been proposed that eye movements in search and free viewing act to maximize information, and are thus guided not just by the most distinctive visual features but by an optimal strategy of uncertainty reduction (e.g. [13–15]). Given the rapid drop-off in resolution away from the fovea, and the average size of saccades (around 4° or 5° in scene viewing [16]), there may be a trade-off between investigating partially visible features and moving to an entirely different part of the visual field.

In distinction to approaches that record participant selections (e.g. fixation sequences) and then measure the properties of selected regions, naturalistic orienting can also be addressed by manipulating the availability of information, and examining the influence of these manipulations on selection. For example, Foulsham *et al*. [17] examined how gaze deployment is influenced by the use of asymmetrical gaze-contingent windows during scene viewing. In particular, using windows that are longer along one axis than the other (i.e. horizontal or vertical slots), they were able to make an important comparison between selection of regions where some information was already visible, and selection of regions having no prior information. Eye movements aligned with the long axis of the window were taken as exploiting existing information, because these movements shifted gaze to a region within the window (e.g. when shifting a horizontal slot left or right). By contrast, saccades moving perpendicular to this direction were taken as exploring unknown regions, because they had the effect of revealing more new information (e.g. when shifting a horizontal slot up or down). Those authors reported a consistent bias in eye movements to follow the long axis of the window, suggesting that eye movements are preferentially deployed to resolve details about existing information, rather than to seek out entirely new information.

This core distinction, between samples that improve or augment existing information (e.g. by foveating a peripherally detected object), and samples that provide entirely new information (e.g. by turning around to reorient the visual field through movements of the body, or by reconfiguring the environment to change its occlusion properties), provides a natural test for differences in selection that may exist across orienting effectors. In particular, while eye movements serve to shift detailed visual attention within the visual field, head and trunk movements serve to shift the visual field itself. Commensurate with these roles, we might expect that body movements should be more predisposed to seeking out new information, providing a complementary orienting bias to that seen in eye movements.

Alternatively, it is often assumed that eye movements are an excellent proxy for attentional orienting in general, be it covert or overt. For instance, the prevailing premotor theory holds that covert orienting is subserved by preparing to execute an eye movement [18–20]. In this light, we might expect that head movements should be little different from eye movements insofar as their selection characteristics are concerned. Indeed, it has routinely been observed that eye and head movements are tightly and systematically coordinated, both in low-level neural circuits (in cats [21,22] and in primates [23,24]), and in behaviour during attentional shifts (e.g. [25,26]). Although top-down influences can override these coordinated behaviours (e.g. [27,28]), the apparent ‘default’ pattern suggests that head- and eye-based attentional systems orient most often using common or shared general principles.

**Figure 1.**
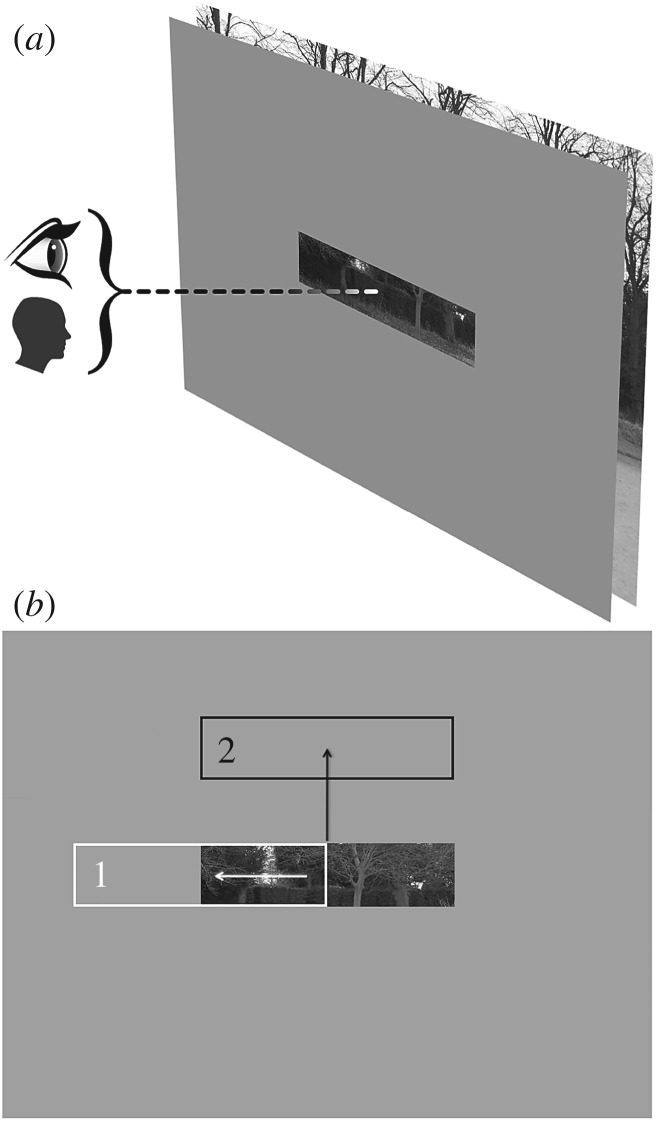
(*a*) In gaze-contingent displays, an opaque mask obscures the image, with the exception of a ‘window’, whose position is determined moment to moment by the recorded position of the eye or the head. (*b*) In asymmetrical windows, we differentiate between gaze shifts aligned with the long axis of the window, which result in inspection of areas where information is already available (1), and gaze shifts aligned perpendicular to the long axis, which result in inspection of areas where no information is currently available (2).

In this study, we evaluate the extent to which orienting behaviours using different effectors may exhibit differential selection characteristics and biases. In particular, we combine the methods of Foulsham *et al.* [17], and Solman & Kingstone [9] to evaluate scene viewing with asymmetric gaze-contingent windows, controlled either by the eyes or by the head. Participants viewed scenes for 10 s and were given a simple yes/no question about the content following each scene (e.g. ‘Was there a red chair?’). There were four viewing conditions and two orienting effector conditions. Participants scanned the scene either in full view, or through an effector-contingent window, which displayed only a portion of the scene at the moment-to-moment position indicated by the effector (figure 1). Effector-contingent windows were either square, horizontal slots or vertical slots. Effector conditions were eye contingent and head contingent, with the head-contingent window condition (conceptually equivalent to viewing a dark room with a headlamp) following the methods in Solman & Kingstone [9].

The key comparisons concerned how effector condition would interact with window condition. In particular, whether head movements would, like eye movements, follow the long axis of asymmetrical windows, or instead shift the window in the direction of the short axis, so as to reveal more new information, that could then be inspected by the eyes.

## 2. Material and methods

### 2.1. Participants

In total, 40 participants (20 per effector condition; nine male, 31 female) from the University of British Columbia participated for course credit. All participants reported normal or corrected-to-normal visual acuity. Informed consent was obtained from all participants, and all experimental procedures and protocols were reviewed and approved by the University of British Columbia Behavioral Research Ethics Board.

### 2.2. Displays and procedure

Displays subtended 39.2° by 29.4°, and windows were 15.3° by 3.8° for the asymmetrical windows, and 7.7° for the square window. Each participant viewed the same 40 scenes in randomized order, with window condition blocked and counterbalanced (10 scenes per window condition). Each trial began with a black dot displayed on a grey background, in one of the four corners or in the centre of the display. Participants shifted their gaze onto the dot to trigger onset of the image—in this way ensuring that gaze-shift patterns did not reflect stereotyped patterns specific to a central starting position. Images were viewed for 10 s, followed by a yes/no question about the content following each scene (e.g. ‘Was there a red chair?’) in order to motivate visual exploration of the images. Participants responded via keypress (‘z’ or ‘m’) with yes–no mapping counterbalanced across individuals. No feedback was provided, and the following trial began immediately after the participant's response. Participants completed two practice trials with a square window to familiarize themselves with the task, and a short break and recalibration occurred between each window condition.

### 2.3. Tracking methods

Native data rates were 1000 Hz for the eye-contingent apparatus and 100 Hz for the head-contingent apparatus. For both effector conditions, we performed temporal smoothing of detected positions to reduce jitter in the displayed window position. Both raw (unsmoothed) and displayed (smoothed) coordinates were stored for analysis at a rate of 60 Hz. Approximate latency differed across systems (approx. 2 ms for the eye-contingent condition and approximately 10 ms for the head-contingent condition), but was in both cases lower than the screen refresh rate (60 Hz).

### 2.4. Apparatus: eye contingent

The experiment was created in Matlab, using v. 3 of the Psychophysics Toolbox [29,30] and the Eyelink Toolbox [31], and run on an SR Research supplied host PC with a 2.67 GHz Intel Core2 Quad CPU. The stimulus displays were presented on a 17^″^ Dell P2411Hb monitor at a resolution of 1280 by 960 pixels and a distance of 55 cm. Eye movements were recorded throughout the task using a desktop-mounted Eyelink 1000 system (SR Research), with participants' heads stabilized by a chin and forehead rest. Velocity and acceleration were calculated online through SR Research's system, and saccades were identified by a velocity threshold of 30° s^−1^ and an acceleration threshold of 8000° s^−2^, as well as a motion threshold of 0.15°. Saccade offset was registered when these thresholds were no longer surpassed.

### 2.5. Apparatus: head contingent

The experiment was created in Matlab, using version 3 of the Psychophysics Toolbox, and run on a Dell Precision T3500 computer with a 3.07 GHz Intel Xeon Processor. Stimulus displays were rear-projected with a Canon LV8235-UST projector onto a Da-Lite screen with a diagonal span of 132^″^ at a resolution of 1920 by 1080 pixels and a distance of 300 cm. Note that, to match the aspect ratio of the monitor in the eye-contingent apparatus, we used only a restricted horizontal span of the projection screen for image display, so that in practice the resolution used was 1440 by 1080 pixels, and the diagonal span commensurately reduced. Head position was recorded using an OptiTrack optical motion tracking system (Natural Point, Inc.) with six V100:R2 cameras. The participant's head was tracked using a rigid body (providing 6DOF position and orientation) defined by five passive reflection markers, affixed to the front of a baseball cap. The position and orientation of the head was then used to determine the window position on the screen, as if the participant was wearing a headlamp.

### 2.6. Analysis

Analyses were based on the angular distribution of gaze movements during scene viewing (example scanpaths are shown in electronic supplementary material, figure S1). Each eye or head movement was classified on the basis of its onscreen angle (i.e. for the vector from the start to the end position of the movement), and the proportion of movements within each of 16 equal-sized (*π*/8) bins was determined. For eye movements, we examined the distribution of saccade directions. For head movements, which are not ballistic in the way that saccadic eye movements are, we estimated the distribution using a sample-based approach. In particular, for each sample position *p_n_* = (*x_n_*, *y_n_*), we stepped forward through successive samples (*n* + 1, *n* + 2, … , *n* + *k*) until the magnitude of the vector from *p_n_* to *p_n_* _+_ *_k_* exceeded 1.0 degrees of visual angle (equivalent to a fairly short saccade; see electronic supplementary material, figure S2).

We then calculated the horizontal bias of these distributions—i.e. the proportion of movements oriented horizontally. For both eye and head movements, vector angles were classified as horizontal ({0 ± 3*π*/16} ∪ {*π* ± 3*π*/16}), vertical ({*π*/4 ± 3*π*/16} ∪ {3*π*/4 ± 3*π*/16}), or neither (these vectors were discarded). Horizontal bias was calculated as the proportion of horizontally classified vectors divided by the sum of both horizontal and vertical categories.

Finally, these bias scores were used to determine an axis congruency measure—i.e. the degree to which movements align themselves with as opposed to against the long axis of asymmetrical windows. In particular, we take the average horizontal bias in horizontal windows and the average vertical bias in vertical windows. These two values are combined, and the mid-point (0.5) is subtracted, to yield positive scores when movements are biased to align with the asymmetrical window, and negative scores when movements are biased in the orthogonal direction.

## 3. Results

**Figure 2.**
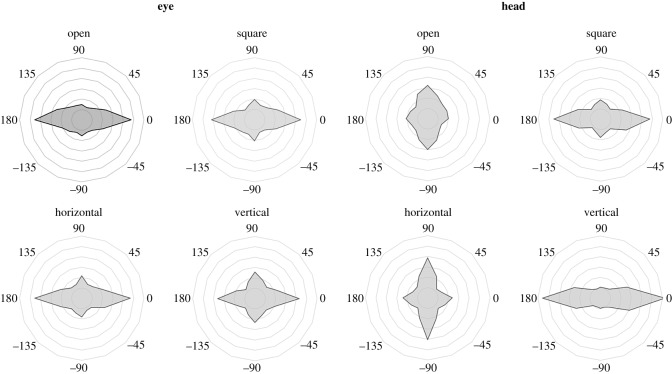
Angular distribution of gaze movements during scene viewing for eye and head movements with open (no window), square, horizontal and vertical windows, plotting the proportion of gaze shifts across angles. Rings are spaced in 3% increments, to a maximum of 18% at the outer ring.

**Figure 3.**
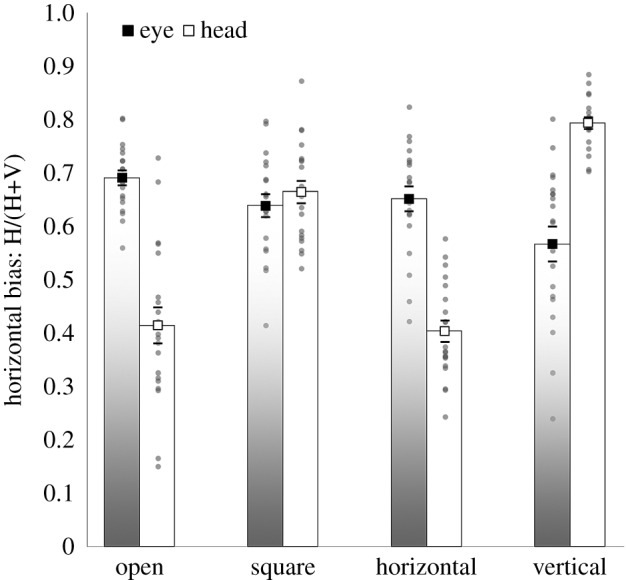
Horizontal bias scores for eye and head movements for gaze-contingent viewing with open (no window), square, horizontal and vertical windows. Grey circles show individual data points and square markers indicate mean values. Error bars depict one standard error of the mean.

Angular distributions of movements during scene viewing are plotted in figure 2, for each effector and each viewing condition. The horizontal bias scores in these distributions (see Analysis in Material and methods) are shown in figure 3, and were analysed with an effector (eye, head; between subjects) by window (open, square, horizontal, vertical; within subjects) mixed factors ANOVA. There was a main effect of window, *F*_3,114_ = 26.9, m.s.e. = 0.008, *p* < 0.001, a main effect of effector, *F*_1,38_ = 9.72, m.s.e. = 0.019, *p* < 0.005, and, critically, a significant effector by window interaction, *F*_3,114_ = 69.8, m.s.e. = 0.008, *p* < 0.001. This interaction was resolved by conducting a separate repeated measures ANOVA for each effector condition, with window (open, square, horizontal, vertical) as the sole factor.

Both conditions had significant effects of window, eye contingent: *F*_3,57_ = 6.59, m.s.e. = 0.008, *p* < 0.001, and head contingent: *F*_3,57_ = 89.5, m.s.e. = 0.008, *p* < 0.001. These effects were followed up with targeted contrasts, first comparing each of the asymmetrical windowed conditions to the baseline square window condition,^1^ then additionally directly comparing horizontal and vertical window conditions.

For the eye-contingent window, there was no difference in horizontal bias between the horizontal window and the square window, *F*_1,19_ < 1, *p* = 0.648, but a significantly reduced horizontal bias (i.e. more vertical movements) for the vertical window when compared with the square window, *F*_1,19_ = 6.44, m.s.e. = 0.016, *p* < 0.05. Most critically, there was a significantly greater horizontal bias in the horizontal window when compared with the vertical window, *F*_1,19_ = 5.84, m.s.e. = 0.025, *p* < 0.05, replicating the key finding in [17]. When viewing with an eye-contingent horizontal window, the majority of saccades were horizontal. When viewing with a vertical window, a higher proportion were vertical. Thus, rather than moving the window to show more information, they tended to target locations that were already revealed.

For the head-contingent condition, there was both a reduced horizontal bias for horizontal when compared with square window conditions, *F*_1,19_ = 123.0, m.s.e. = 0.011, *p* < 0.001, and an increased horizontal bias for vertical when compared with square window conditions, *F*_1,19_ = 66.1, m.s.e. = 0.005, *p* < 0.001. Finally, directly comparing the asymmetrical windows, we find a greater horizontal bias for vertical when compared with horizontal window conditions, *F*_1,19_ = 492.6, m.s.e. = 0.006, *p* < 0.001. In other words, head-contingent participants showed the complete opposite effect of asymmetrical window condition to the eye-contingent group. The majority of head movements elicited by a horizontal window were vertical, exposing more new information. With a vertical window, this switched systematically to a horizontal bias.

### 3.1. Axis congruency

**Figure 4.**
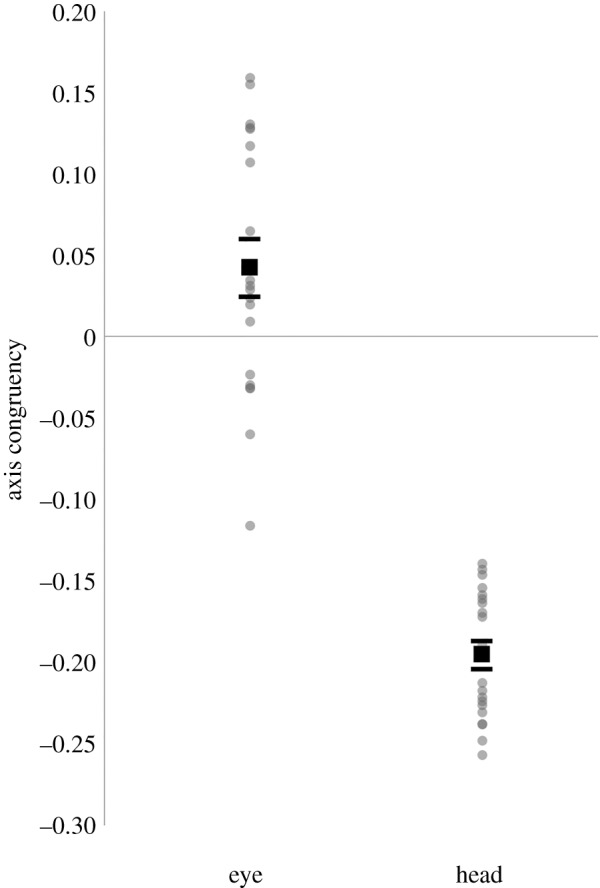
Axis congruency scores for eye and head movements with asymmetrical gaze-contingent windows. Positive scores indicate greater than chance alignment with the window's long axis. Negative scores indicate greater than chance alignment perpendicular to the window's long axis. Grey circles show individual data points and square markers indicate mean values. Error bars depict one standard error of the mean.

A more targeted measure to assess the influence of asymmetrical windows is to compute the average axis congruency of orienting movements—i.e. the degree to which movements align themselves with as opposed to against the long axis of asymmetrical windows (see Analysis in Material and methods). Both effector conditions showed significant bias (figure 4), confirmed with two-tailed single-sample *t*-tests against 0 (i.e. against the assumption of no bias). For eye-contingent viewing, there was a significant positive bias, *t*_19_ = 2.417, *p* < 0.05, with more movements along the main axis of asymmetrical windows. For head-contingent viewing, there was instead a significant negative bias, *t*_19_ = −22.194, *p* < 0.001, with more movements against the main axis of asymmetrical windows.

## 4. Discussion

We sample our visual environment in multiple ways, by moving our bodies, heads and eyes. Despite this, we know rather little about how these different movements are deployed and coordinated to explore the world. This study provides good evidence that, rather than different types of movements being driven by the same underlying guidance processes, overt attentional orienting is guided differently based on the particular effector being used.

The different windowed viewing conditions in this work resulted in clear and effector-specific changes in behaviour. When the window was yoked to *eye position*, overall slightly more horizontal than vertical saccades were made, a bias typical to eye movements and reported previously [32]. Critically, when viewing through asymmetrical windows, this bias was modulated in the direction parallel to the long axis of the window—with vertical windows leading to a reduction of the typical horizontal bias, so that participants more often shifted their gaze up and down within the window. In other words, participants moved their eyes to exploit the currently visible information more often than making a saccade into an unseen region (cf. [17]). This pattern is the opposite to what one would expect if the eyes are moving to reveal new information [33], which is surprising for some models of eye movement control (e.g. [14]).

When viewing was instead controlled by *head position*, however, the core pattern of results was entirely reversed. Horizontally oriented windows led to an increase in vertical saccades, and vertically oriented windows resulted in far more horizontal saccades. In these conditions, therefore, participants tended to explore the scene by moving perpendicular to the long axis of the window, resulting in new information becoming visible. This information could then be exploited by making eye movements within the window. Critically, even though the windows restricted visibility in exactly the same way for the head-contingent and eye-contingent conditions, participants oriented their attention differently according to the effector involved.

Notably, the magnitude of the orienting bias for asymmetrical windows was considerably larger for the head than for the eyes. This may suggest that eye movements are less readily influenced by top-down or strategic factors than are slower and more deliberate head movements, so that the underlying horizontal bias in eye movements (independent of asymmetrical viewing restrictions) remains prominent across conditions. This default horizontal bias may reflect a combination of asymmetry in the visual field, the side-by-side placement of the eyes, and priors on information localized along the horizon. The vertical window, then, attenuates this bias but does not remove it. Given that inhibition of reflexive eye movements is notoriously difficult (e.g. antisaccade tasks; [34]), it is perhaps unsurprising that typical biases in eye movements are more resistant to experimental manipulation than are head movements.

The interaction between window shape and effector reported here provides some key insights into attentional selection in systems, such as the human visual system, which contain nested effectors for orienting. In particular, our results suggest that while eye movements may be biased to target regions of the visual field where there is some pre-existing information, head movements are instead biased to target unknown regions. This distinction provides a fundamental complementarity in nested orienting systems, so that coarse orientation provides entirely novel information, while fine orientation more selectively scrutinizes existing information. When considering how people scan scenes, several authors have made the distinction between large global shifts which change the focus to a new and unknown region and smaller local movements which focus on detailed inspection [33,35]. In order to see what we need to see, a balance must be struck between exploration and exploitation. Our results suggest that there is a division of labour in this regard between the eyes and the head, and presumably the rest of the body.

Why do the eyes and head respond in this way to the presence of asymmetric windows? It is important to note that in unrestricted viewing, humans have a wider field of view and greater resolution horizontally than vertically [1]. In effect, the conditions of naturalistic viewing are themselves asymmetric along the horizontal axis. Commensurately, we note here that the open (no window) and horizontal windows produced the most similar behaviours, in both eye- and head-contingent conditions. In the case of eye movements, a horizontal bias was observed (as noted elsewhere [32]), and in the case of head movements, a vertical bias was observed. Thus, under unrestricted viewing conditions, we find eye movements preferentially exploiting the greater horizontal span of the visual field, while head movements serve the role of shifting this field vertically. When the horizontal range is restricted—through square or vertical windows—these orienting systems accommodate. Under eye-contingent viewing, the typical horizontal bias is attenuated, with more gaze shifts drawn into alignment with the window, following the visual information presented there. Under head-contingent viewing, the eye is no longer able to drive horizontal exploration, and so the head assumes this role.

A similar scenario has been explored in the context of reading [36]. Head and eye movements were recorded while participants read a passage on either a vertically or horizontally extended page. In this case, the eyes were found to exploit information both horizontally and vertically, but with some attenuation along the longer axis. Head movements, on the other hand, supplemented this eye-based exploration with increased vertical scanning for vertically arranged material, and increased horizontal scanning for horizontally arranged material. These results may at first seem contradictory, but it is important to emphasize the difference between asymmetry in the environment and asymmetry in the ability to inspect the environment. In the former case, there is by definition no information to be had outside of the proscribed area—if the information is itself extended along one axis, there is necessarily nothing to be learned from exploration orthogonal to that axis. By contrast, if information is distributed uniformly, but *visibility* is restricted to one axis, then shifts orthogonal to that axis may prove useful. An additional key distinction concerns the goals which drive the visual system. In the reading study [36], the sequence of gaze positions was determined by the task, and with the exception of line changes on the horizontally oriented page, successive words were always within the visual field. Here, then, head movements were recruited solely to reduce the oculomotor strain from eccentric eye positions, or to support shifts too large for the eyes alone. In this study, the task is explicitly exploratory, and although salient points in a scene may preferentially capture attention, there is nothing like the regularity of sequential fixations seen in reading, nor is there strong systematicity in salient points across different images. In this exploratory setting, we find that the head is far more proactive—guided in such a way as to uncover new information to a greater extent than movements of the eyes under the same conditions.

This complementarity between effectors probably optimizes the utility of foveal attention. In eye movements alone, there have been suggestions that fixation locations are selected to maximize uncertainty reduction—targeting regions of the visual field probably to provide the most new information [13–15]. Importantly, however, these selections are all made in the presence of peripheral vision in those regions. In other words, information-maximizing eye movements select on the basis of existing coarse visual information. Shifting attention to a region entirely outside of the immediate visual field, however, necessitates foveating an arbitrary and completely unknown region—which may often have little informational value (e.g. a patch of clear sky or a blank wall). To engage saccadic targeting processes for such a gaze shift is probably a waste of resources. Head movements may therefore serve an important bridging role, shifting the visual field at a coarse level, then allowing eye movements to select the important regions of this new vista.

It is important to note that this account, at face value, seems contradictory with reliably observed patterns of coordination between eye and head movements during large gaze shifts—with the eye characteristically *leading* or else synchronous with the head in movement onset (e.g. [4,25,26]). Based on these results, our account seems untenable—it is difficult to argue that the head's role is to provide a novel field for the eye if head movements characteristically lag behind eye movements. Critically, however, this characteristic pattern is observed only for gaze-shifts to unpredictable targets within the visual field. By contrast, head movements do lead the eyes when targets are predictable (e.g. [25,37]) and when shifts are voluntary (i.e. without a target [38]), suggesting that the temporal coordination between eye and head movements is fundamentally different when gaze shifts are reactive (e.g. to a target stimulus) than when they are intentional. In exploratory orienting, it is the observer driving the selection of gaze locations, not a discrete target stimulus. Consequently, consistent with our account, we would expect the intentional mode of coordination, with the head leading the eyes. Combining these two orienting systems, then, efficient and thorough visual exploration can be achieved. Eye movements are tasked with analysing in detail only the most important regions, conserving foveal processing, driven by *informed* selection within the existing visual field. Head and body movements, on the other hand, provide novel information by selecting the visual field itself, with an exploratory bias towards the unknown.

## Supplementary Material

Two supplementary figures are included, comprising example scanpaths, and a pictorial depiction of our head-contingent direction measure

## Footnotes

1 We use the square window condition as our baseline instead of the open/no-window condition because although eye movements are expected regardless, head movements are not guaranteed in the open condition. The square window ensures exploratory movements in both conditions and provides the critical symmetry control.

## Data accessibility

Our data are deposited at the Dryad Digital Repository: http://dx.doi.org/10.5061/dryad.33kh1 [39].
