# Supplementary material for: Eye and head movements are complementary in visual selection
Source: R Soc Open Sci. 2017 Jan 18;4(1):160569. doi: 10.1098/rsos.160569 (PMC5319320; doi:10.1098/rsos.160569)

*Figure S1.* Example scanpaths for head- and eye-contingent conditions.


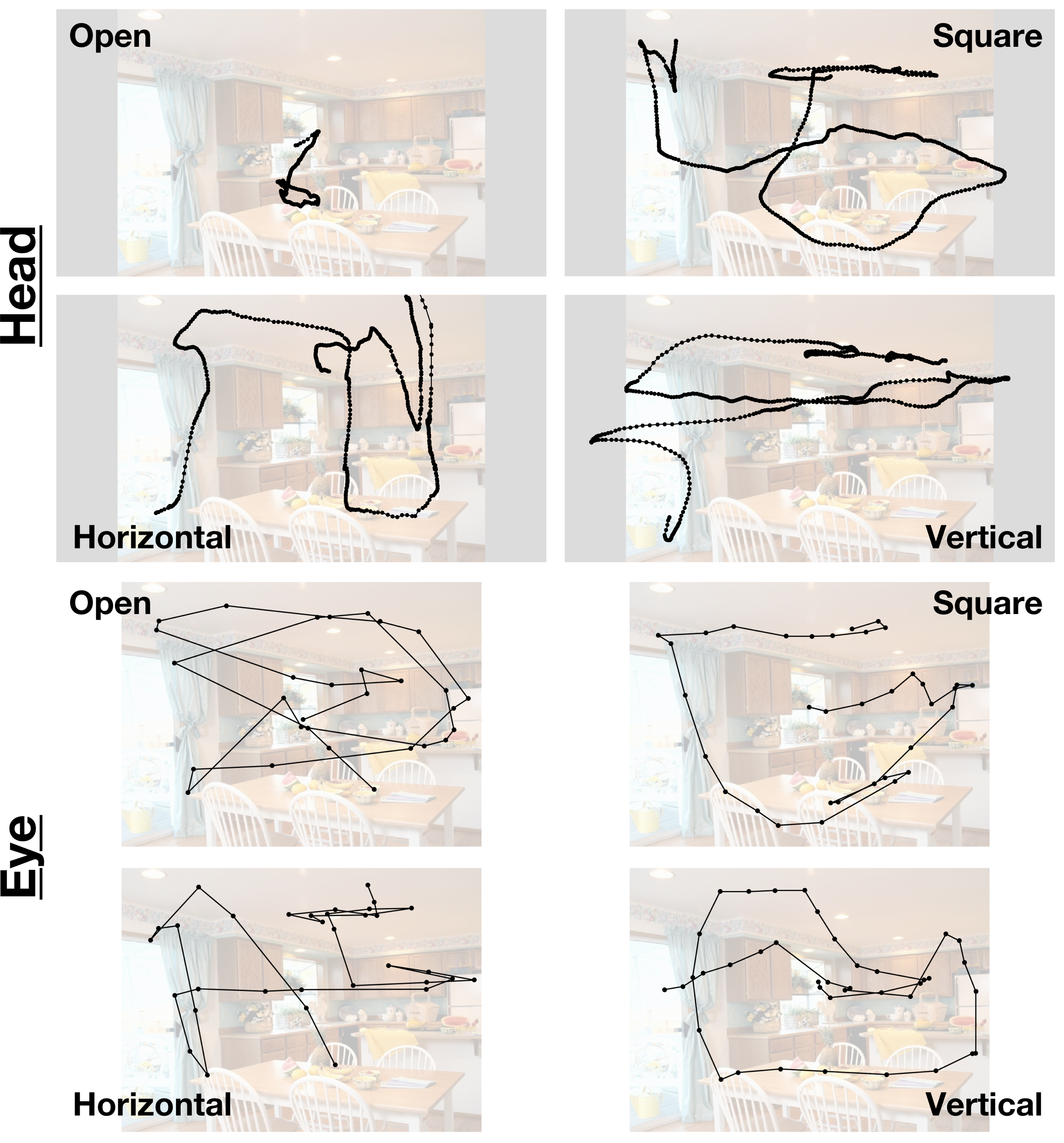


*Figure S2.* Schematic representation of head-movement direction computation. At each sample, p_n_, successive samples (moving forward in time) were inspected until a sample p_n+k_ fell outside of a radius of 1.0 degrees of visual angle (red circle). The vector from p_n_ to p_n+k_ (blue arrow) was used to assign a direction to that sample. In this way, the random noise of small movements (like those of the eye during a fixation) are excluded, while making minimal assumptions about characteristic speed of movement or typical dwell times during head-based exploration.


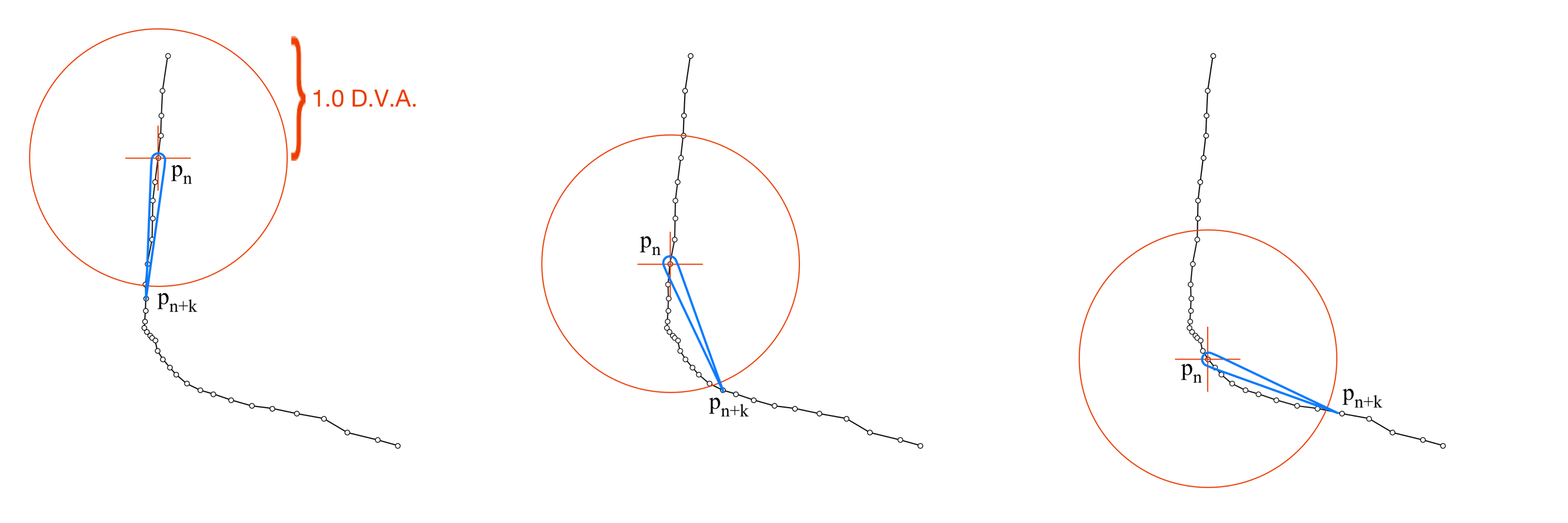

Supplement: Two supplementary figures are included, comprising example scanpaths, and a pictorial depiction of our head-contingent direction measure [file rsos160569supp1.docx]
